# Supplementary figures and images for: Time-series analysis of meteorological factors and emergency department visits due to dog/cat bites in Jinshan area, China
Source: PeerJ. 2024 Jan 18;12:e16758. doi: 10.7717/peerj.16758 (PMC10800098; doi:10.7717/peerj.16758)

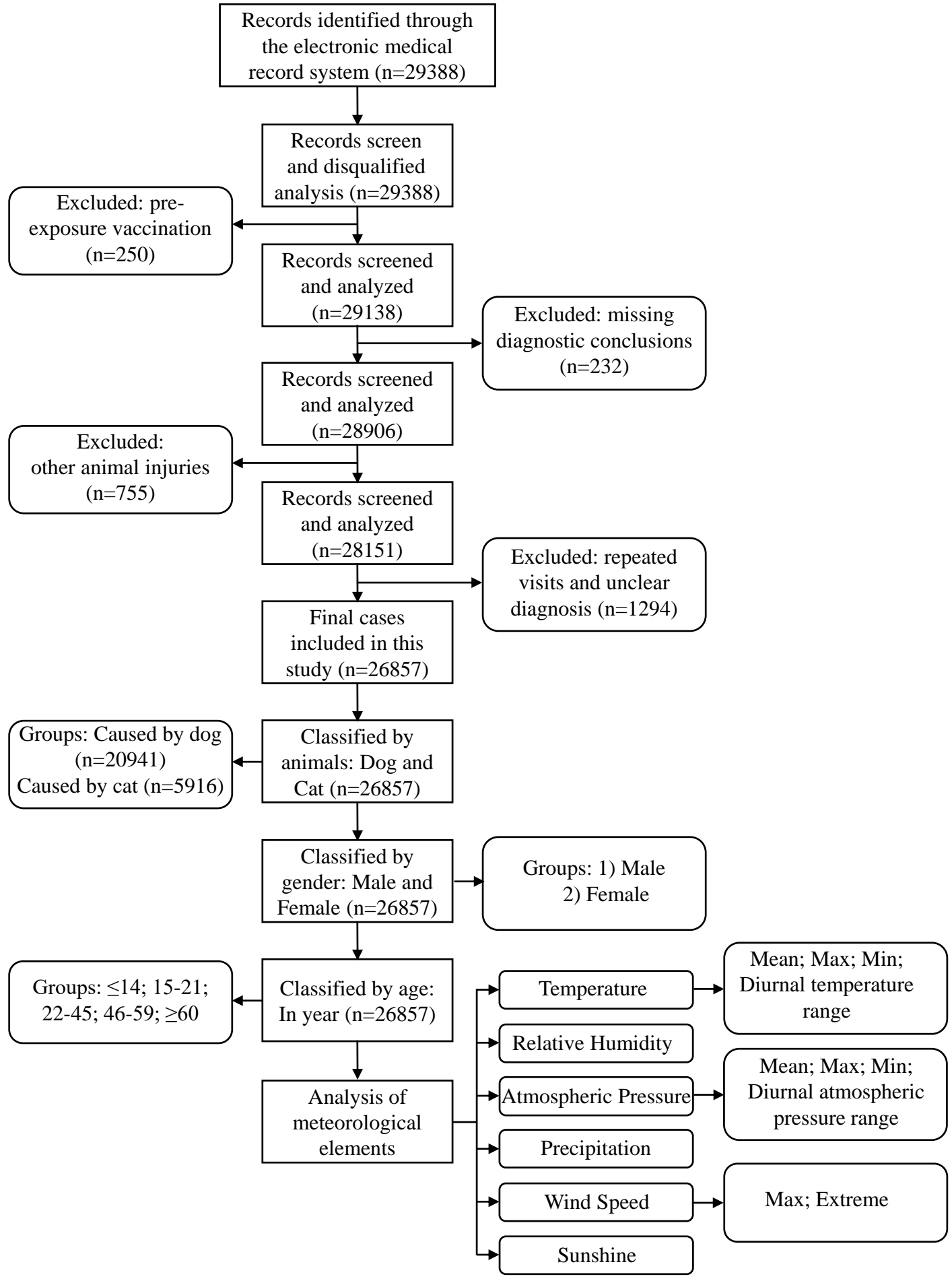

Supplement: Supplemental Information 2 [file peerj-12-16758-s002.pdf]

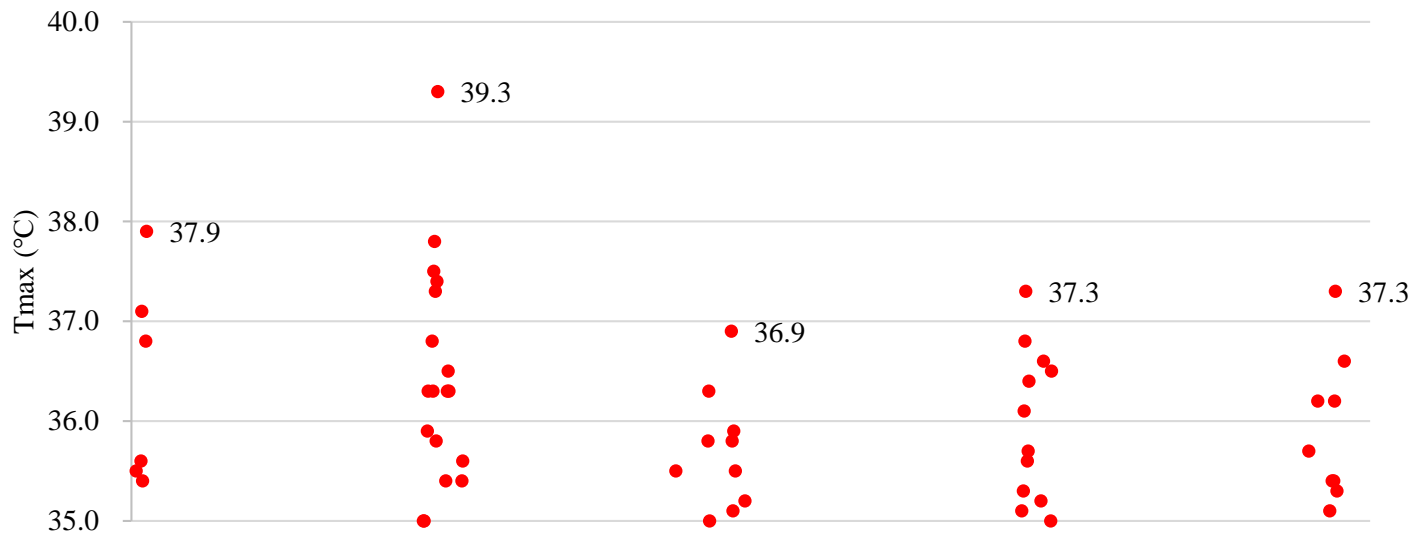

Supplement: Supplemental Information 3 [file peerj-12-16758-s003.pdf]

**A All ages**

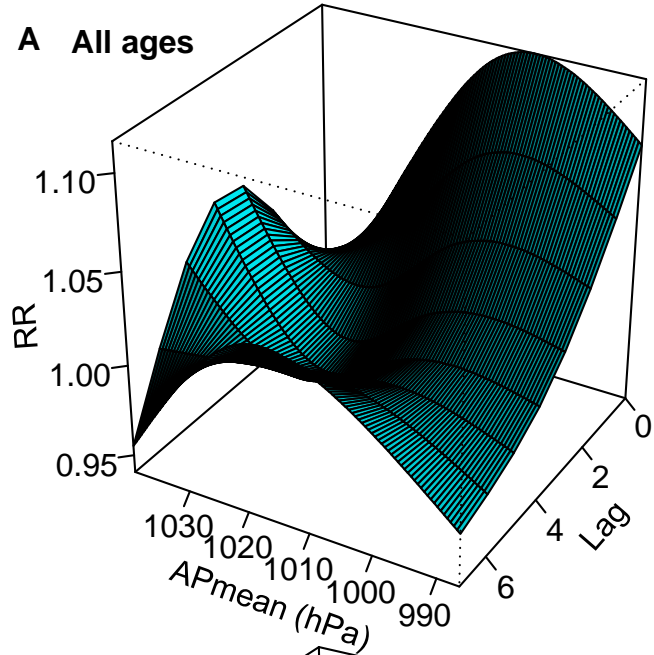

**B 0-14 years**

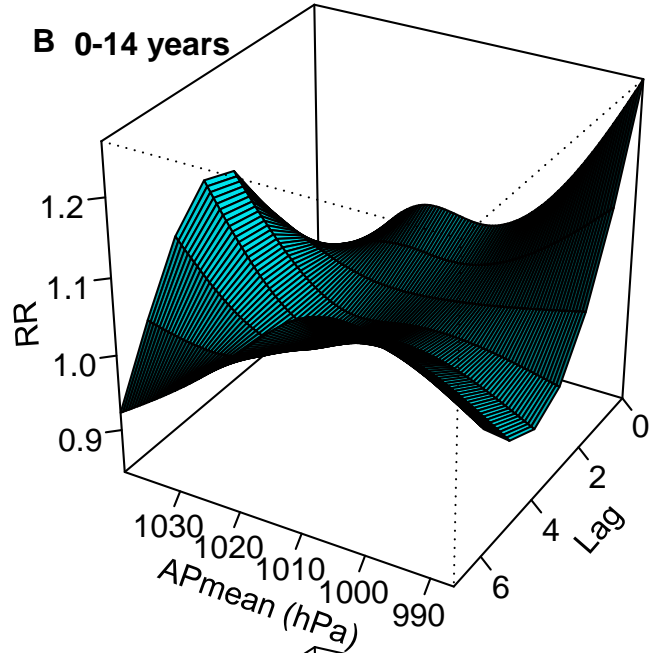

**C 15-21 years**

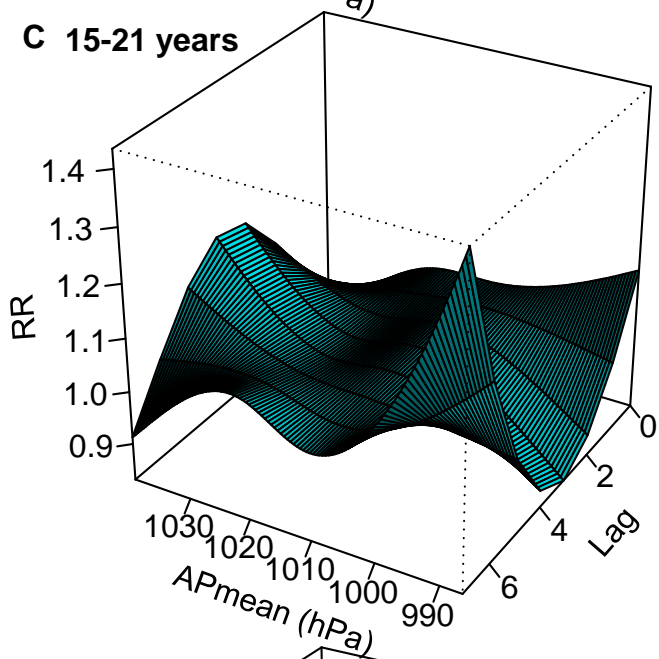

**D 22-45 years**

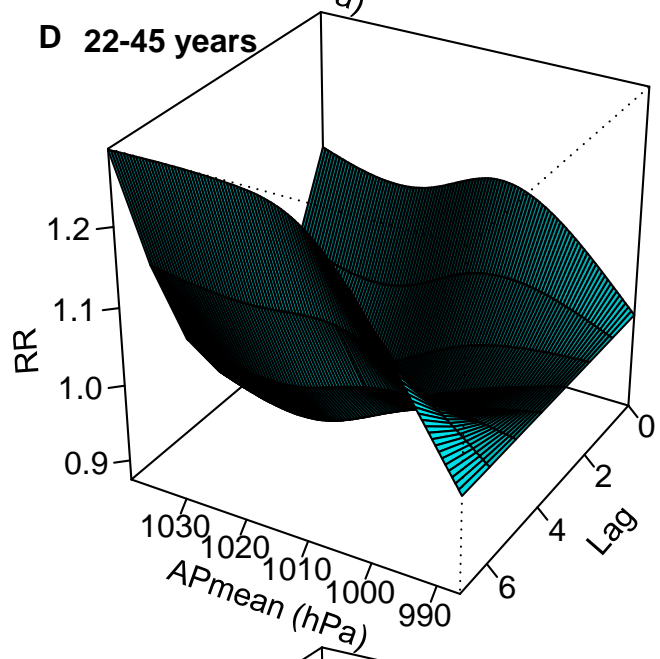

**E 46-59 years**

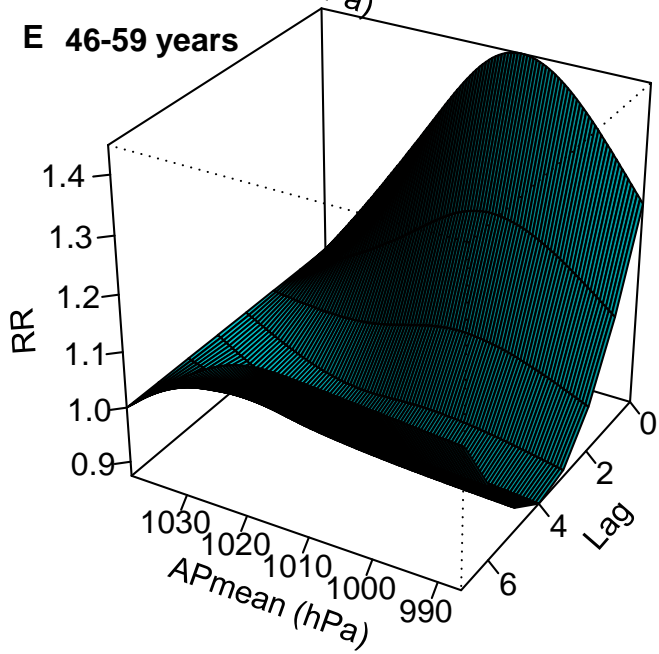

**F  $\geq 60$  years**

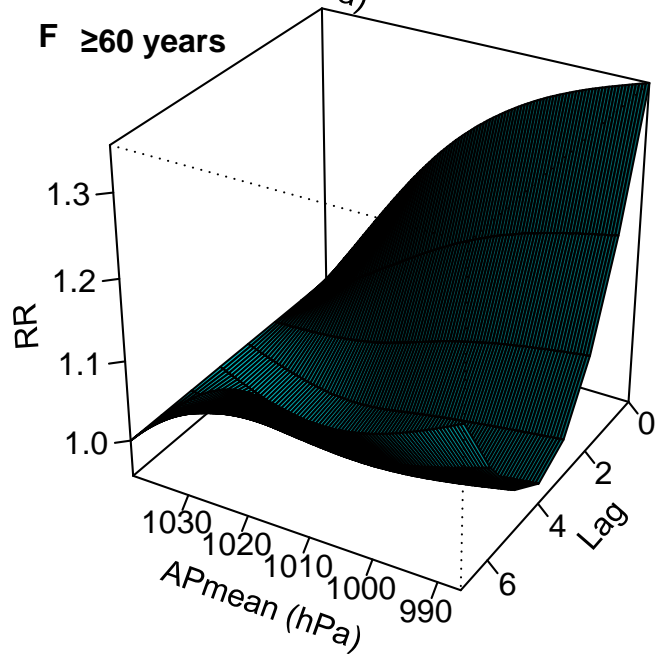

Supplement: Supplemental Information 4 [file peerj-12-16758-s004.pdf]
