# Supplementary material for: Time-series analysis of meteorological factors and emergency department visits due to dog/cat bites in Jinshan area, China
Source: PeerJ. 2024 Jan 18;12:e16758. doi: 10.7717/peerj.16758 (PMC10800098; doi:10.7717/peerj.16758)
Supplement: Supplemental Information 5 [file peerj-12-16758-s005.docx]

**Table S1.** Five-year general demographic information of patients with dog and cat bites

| Variable | Case number (%) |
| --- | --- |
| Sex |  |
| Male | 13,085 (48.72) |
| Female | 13,772 (51.28) |
| Age |  |
| ≤14 | 4,419 (16.45) |
| 15-21 | 2,180 (8.12) |
| 22-45 | 10,305 (38.37) |
| 46-59 | 5,633 (20.97) |
| ≥60 | 4,320 (16.09) |
