# Supplementary material for: Time-series analysis of meteorological factors and emergency department visits due to dog/cat bites in Jinshan area, China
Source: PeerJ. 2024 Jan 18;12:e16758. doi: 10.7717/peerj.16758 (PMC10800098; doi:10.7717/peerj.16758)
Supplement: Supplemental Information 6 [file peerj-12-16758-s006.docx]

**Table S2.** Association of dog and cat bites with high temperature

| Years | Ht (day) | Ht (month) | Mht (℃)±SD | Ht DCBs  Total n (n per day) | Annual DCBs  Total n (n per day) |
| --- | --- | --- | --- | --- | --- |
| 2016 | 6 | July | 36.38 ± 1.034 | 97 (16.2) | 4573 (14.8) |
| 2017 | 18 | July, August | 36.44 ± 1.105 | 429 (23.8) | 5730 (15.8) |
| 2018 | 10 | May, June, July, August | 35.70 ± 0.581 | 176 (17.6) | 5370 (14.7) |
| 2019 | 12 | July, August | 35.97 ± 0.756 | 257 (21.4) | 5749 (15.7) |
| 2020 | 9 | July, August | 35.91 ± 0.722 | 161 (17.9) | 5435 (12.5) |

Note: Ht, high temperature; Mht, mean of high temperature. DCBs, dog and cat bites.
